# Bilateral transtibial amputation with concomitant thoracolumbar vertebral collapse in a Sichuan earthquake survivor

**DOI:** 10.1186/1749-799X-5-43

**Published:** 2010-07-14

**Authors:** Caroline Ngar-Chi Wong, Joseph Man-Kit Yu, Sheung-Wai Law, Herman Mun-Cheung Lau, Cavor Kai-Ming Chan

**Affiliations:** 1 Physiotherapy Department, Prince of Wales Hospital, Shatin, Hong Kong Special Administrative Region, PR China; 2 Prosthetic and Orthotic Department, Prince of Wales Hospital, Shatin, Hong Kong Special Administrative Region, PR China; 3 Department of Orthopaedics and Traumatology, Prince of Wales Hospital, Shatin, Hong Kong Special Administrative Region, PR China

## Abstract

The devastating earthquake in Sichuan, China on 12 May 2008 left thousands of survivors requiring medical care and intensive rehabilitation. In view of this great demand, the Chinese Speaking Orthopaedic Society established the "Stand Tall" project to provide voluntary services to aid amputee victims in achieving total rehabilitation and social integration. This case report highlights the multidisciplinary rehabilitation of a girl who suffered thoracolumbar vertebral collapse and underwent bilateral transtibial amputation. The rehabilitation team was involved in all stages of the care process from the pre-operative phase, through amputation, into prosthetic training, and during her life thereafter. Despite this catastrophic event, early rehabilitation and specially designed bilateral prostheses allowed her a high level of functional ability. The joint efforts of the multidisciplinary team and the advancement of new technology have revolutionized the care process for amputees.

## Introduction

A 7.9 magnitude earthquake struck Sichuan Province of China on 12 May 2008. It was the most damaging natural disaster since the devastating Asian Tsunami of 2004. The earthquake in Sichuan left over 70,000 dead, about 20,000 missing, more than 200,000 injured and almost ten million homeless [1]. The majority of the injured survivors suffered musculoskeletal trauma - often relating to crush injuries - resulting in unilateral, bilateral or even multiple limb amputation, fractures and spinal cord injuries. Long-term and well planned rehabilitation after the acute management is vitally important to maximize their functional states and rebuild their lives [2-4].

In view of the great demand for medical care and rehabilitation for these victims, the Chinese Speaking Orthopaedic Society established the "Stand Tall" project with the objective to provide voluntary medical and rehabilitative care to those in need. Its ongoing mission is to facilitate and provide comprehensive rehabilitation services for the Sichuan earthquake amputee victims so they may achieve total rehabilitation and social integration. The organization has the belief that all the amputee victims can "Stand Tall" again with self-respect, confidence and social fulfillment [5].

The first operation by the "Stand Tall" program was commenced on 7 June 2008. In less than a month, more than 150 medical professionals were recruited. They included orthopaedic surgeons, nurses, physiotherapists, occupational therapists, and prosthetic and orthotic professionals. The Society collaborated with various Guangdong and Sichuan hospitals to provide an individualized rehabilitation plan and prosthetic fitting for each patient.

In the following article, the authors present a case report of a traumatic bilateral transtibial amputee with concomitant thoracolumbar vertebral collapse in the aftermath of the earthquake, outlining the therapeutic and rehabilitation process.

## Case Background

The patient, a 14-year-old girl living with her family in Sichuan, was a Form 3 student studying in Chui Yuen Secondary School at the time of the earthquake. Her premorbid level of functioning had been independent for all personal daily activities. She was outgoing and actively participated in sporting activities before the incident.

**Figure 1:**
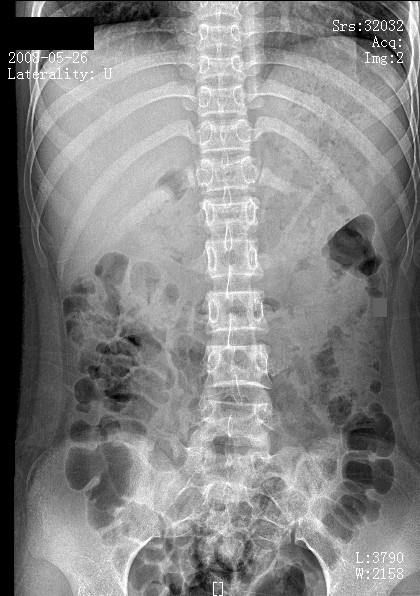
**Pre-operative radiograph (anteroposterior view) with thoracolumbar vertebral collapse**.

**Figure 2:**
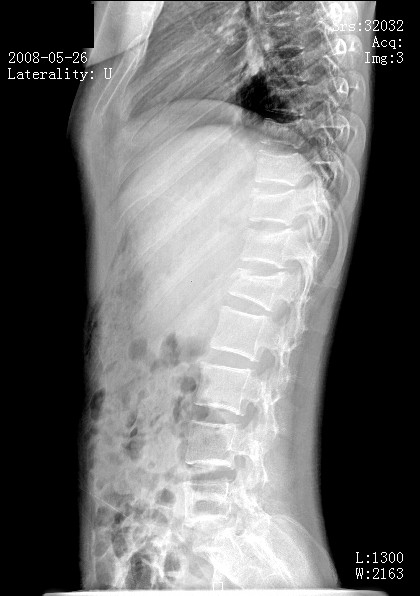
**Pre-operative radiograph (lateral view) with thoracolumbar vertebral collapse**.

**Figure 3:**
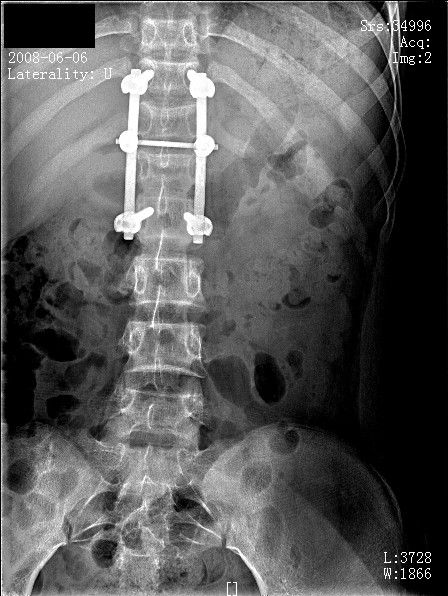
**Post-operative radiograph (anteroposterior view) with posterior spinal fusion performed**.

**Figure 4:**
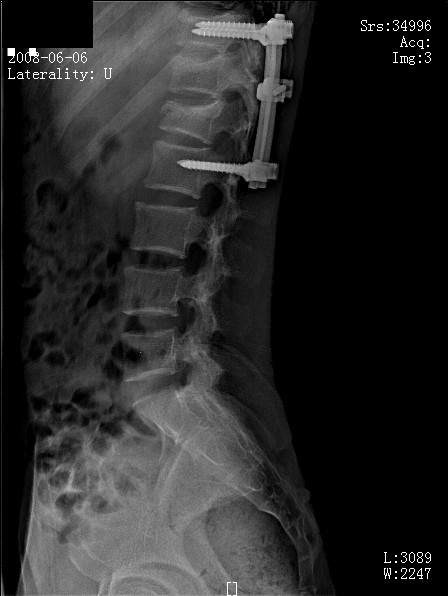
**Post-operative radiograph (lateral view) with posterior spinal fusion performed**.

When the earthquake occurred, the patient was having a lesson at school. Her whole life was dramatically changed in that short period of time. The building collapsed and she was trapped underground for more than 20 hours. She was eventually rescued and sent to a local hospital. However, as she was trapped for so long, her spine and bilateral lower limbs suffered severe injuries. She was diagnosed with thoracolumbar vertebral collapse and both of her legs later became necrotic. A posterior spinal fusion and bilateral below knee amputations were performed on 12 June 2008 (Figures 1, 2, 3, 4).

**Figure 5:**
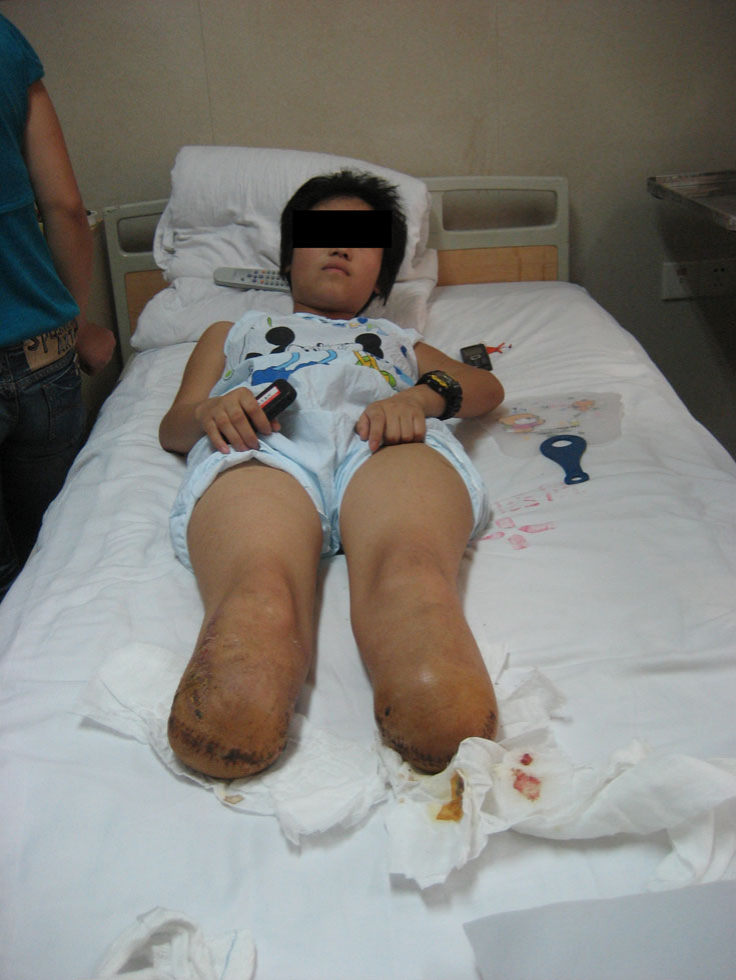
**The patient with bilateral transtibial amputation**.

The first assessment of the patient was performed at Nanfeng Hospital, Guangzhou in June 2008 (Figure 5). The patient was depressed and lacked motivation for exercise training. Her wounds had not yet healed and both stumps remained obviously swollen. She also experienced phantom limb pain and sensitization.

Due to prolonged bed rest, she had developed sacral sores and bilateral hip flexor contractures. Her bilateral lower limbs mobility and power had also decreased. Attributable to pain and weak trunk musculature she was highly dependent on medical staff, requiring manual assistance for bed mobility and transfers. She could neither sit unsupported nor tolerate prolonged supported sitting. This patient then underwent stages of rehabilitation and functional training under the rehabilitation team.

## The Rehabilitation Team

Rehabilitation following amputation is a complex long-term process and is the responsibility of a multidisciplinary team, with the patient focused at the centre. The core members of the rehabilitation team include physicians, nurses, physical therapists, occupational therapists and prosthetic experts. Psychologists, social workers and vocational counselors can also be called in as needed. The patient is at all times considered an active, equal member of the team and has the opportunity to explain his or her needs, preferences, and goals [6,7].

The multidisciplinary team implements comprehensive programs to meet the physical, psychological and functional needs of the client. Different professionals demonstrate competence in areas of expertise in planning and implementation of treatment process. Working with a specialist team produces the best outcome for an individual who has undergone life-changing amputation surgery [8,9].

## Stages of Rehabilitation

The rehabilitation team was involved at all stages of the process, from the pre-operative phase, through amputation, into prosthetic training and during her life thereafter. The course of rehabilitation for this patient was focused into 8 stages namely post-operative, pre-prosthetic, prosthetic prescription/fabrication, prosthetic training, functional training, community reintegration, recreational/vocational rehabilitation, and long-term follow-up.

1. Post-operative: providing emotional support, promoting limbs hygiene and expediting wound healing, maximizing limbs shrinkage and stumps shaping, controlling phantom pain and alleviating phantom sensation

2. Pre-prosthetic: improving joint mobility and muscle strength, facilitating independence and exploring prosthetic options

3. Prosthetic prescription/fabrication: team consensus on prosthetic prescription

4. Prosthetic training: prosthetic management to increase wearing time and functional use

5. Functional training: advanced skills and daily activities training

6. Community reintegration: resumption of family and community roles, developing healthy coping strategies

7. Recreational/vocational rehabilitation: assessment and training for recreational activities, assessment of further education needs or job modification

8. Long-term follow-up: regaining emotional equilibrium, lifelong prosthetic, medical and functional assessment

### 1. Post-Operative Program

Post-operative care was required immediately after surgery. This was a preparatory time for emotional and physical healing. The rehabilitation team executed the following measures in order to accelerate the emotional and physical recovery process.

#### a) Providing Emotional Support

The support team established an ongoing supportive and trusting relationship with the patient and her family to facilitate open discussion. The team was sensitive to her emotional needs at all stages of the rehabilitation process. The patient was introduced to others with similar amputations and comparable circumstances, such as similar levels of amputation or disabilities [10].

#### b) Promoting Limbs Hygiene and Expediting Wound Healing

The patient was instructed to wash the limbs daily with mild soap then dry them thoroughly. Creams were also applied at the suture lines to loosen crust-like formations and expedite wound healing.

#### c) Maximizing Limbs Shrinkage and Stumps Shaping

The goal was to shrink and shape the residual limbs so that they were tapered at the distal end; this allowed for optimal prosthetic fit. Exercises, elevation, intermittent and elastic compression were used to improve the circulation, thereby promoting the healing process, reducing swelling and thus pain. Moreover, tailor-made pressure stump socks or pants made of lycra-net were fabricated to patient for easier handling of her stumps condition.

#### d) Controlling Phantom Limb Pain

Amputation surgery creates tissue disruption and trauma. This produces a natural inflammatory response resulting in oedema. This pressure and injury to the nerve endings causes pain [11]. Phantom pain is described as pain experienced in the missing limb part. It may be intermittent or constant, and can be felt in any part of the removed limb. It is a feature that can impact significantly on the life of a patient [12,13].

Post-operative treatment for this patient with severe phantom pain included analgesics and epidurals. Managing the oedema could aid pain relief. The pain increased with stress. Therefore therapists assessed her pain carefully to determine its cause and allayed her fears to keep stress levels to a minimum. The team members were advised to avoid emphasizing pain whenever possible. Effective pain-relieving techniques/modalities for phantom pain including relaxation, massage, percussion, compression, exercise, acupuncture, ultrasound, transcutaneous electrical nerve stimulation [14,15] and mirror box [16,17] were exploited.

#### e) Alleviating Phantom Limb Sensation

Phantom limb sensation is most common in traumatic amputations. According to Melzack (1989) the neural system related to the missing limb exists within the brain even when the limb is removed by amputation [18]. The following interventions were employed to desensitize the residual limbs so that they would accommodate touch and pressure in preparation for encasement in the sockets.

• Massage was used to desensitize, prevent/release adhesions and soften scar tissue

• Tapping and rubbing the residual limbs and applying a vibrator

• Residual limbs wrapping contributed to desensitizing the limbs

• A desensitization kit made of different textured materials

• The patient put weight on the end of the limbs against various surfaces. These surfaces were graded from very resilient, such as soft foam, to variously resistant and textured, such as layers of felt, rice, and clay. The patient was directed to push the limbs down into the surface for 5-second intervals and increased the contact time and pressure as tolerated

### 2. Pre-Prosthetic Program

The pre-prosthetic therapy program occurred from the post-surgical period until the patient received the permanent prostheses.

#### a) Improving Joint Mobility and Muscle Strength of the Limbs

A physical conditioning regimen should be instituted to maintain or improve the mobility of all joints proximal to the amputation. Mobilization of the limbs also enhanced circulation and reduced oedema. Improving muscle strength of the residual limbs and shoulder areas were also emphasized. For this patient, there was a shift in weight and center of gravity. Regular core strengthening exercises could prevent asymmetry, restore proximal body motion and sense of control [19].

#### b) Facilitating Independence in Daily Activities

Establishing some degree of independence was essential for this patient who had undergone bilateral amputations, and this must be addressed promptly to lessen feelings of dependency and frustration. She was trained to be independent and proficient in managing daily activities [20].

#### c) Exploring Prosthetic Options

Therapists and prosthetic specialists educated the patient about prostheses appropriate to the level of amputation to guide her in establishing realistic expectations. Regular meetings were arranged between the patient and other victims with a similar level of amputation, so that they could talk candidly about any issues of concern, including positive and negative features of prostheses. Factors to be considered when prescribing the prostheses included:

• Residual limbs: skin integrity, length, range of motion and strength

• Preference for cosmetics and function

• Activities at home, school, community and recreational interests

• Motivation and attitude

• Cognitive abilities to learn and use prosthetic controls

### 3. Prosthetic Prescription/Fabrication

The rehabilitation team has come to know the patient in some depth during the pre-prosthetic program regarding her social and cultural contexts. According to the clinical assessment of the amputee and information from therapists, the appropriate prosthetic prescription was determined to match her functional needs [21].

The bilateral below knee prostheses incorporated a Patellar Tendon Bearing socket design and a Flex-foot. The contour of the patellar ligament was utilized as the major weight-bearing surface. The proximal walls of the prostheses extended to the level of adductor tubercle of the femur and provided rotational control and medio-lateral knee stability. A supracondylar suspension with soft insert acted as a relatively simple and effective suspension method.

The CarbonX Active Heel of the Flex-foot stored energy and absorbed shock loads, while the full-length toe lever contributed to stability and even stride length. It provided a normal range of motion and a symmetrical gait. The particular layering of carbon fiber was carefully designed to offer the support and flexibility needed for varied movements. The carbon fiber deflected during heel-off and returned to its resting position during toe-off. The smooth roll of the heel and ankle rocker minimized vaulting, hence improving gait efficiency and reducing energy expenditure.

The positive plaster casts were acquired using a hand-casting method with the patient in an upright sitting position. Using both a frontal and lateral view the alignment was marked on the plaster cast. In the trial fit with the diagnostic socket (made of transparent thermoplastic), several aspects should be checked to ensure a comfortable fit.

The transparent socket was convenient for pressure profiling and volume checking to ensure proper fitting. The proximal trim-line was trimmed down to minimize the hindrance in sitting and walking. The height of a bilateral amputee was depended on the length of the prostheses. In the standing trial, the balance between cosmetic aspects and stability was carefully considered. The orientation of the socket and prosthetic foot complex was adjusted during the evaluation of the static and dynamic alignment.

**Figure 6:**
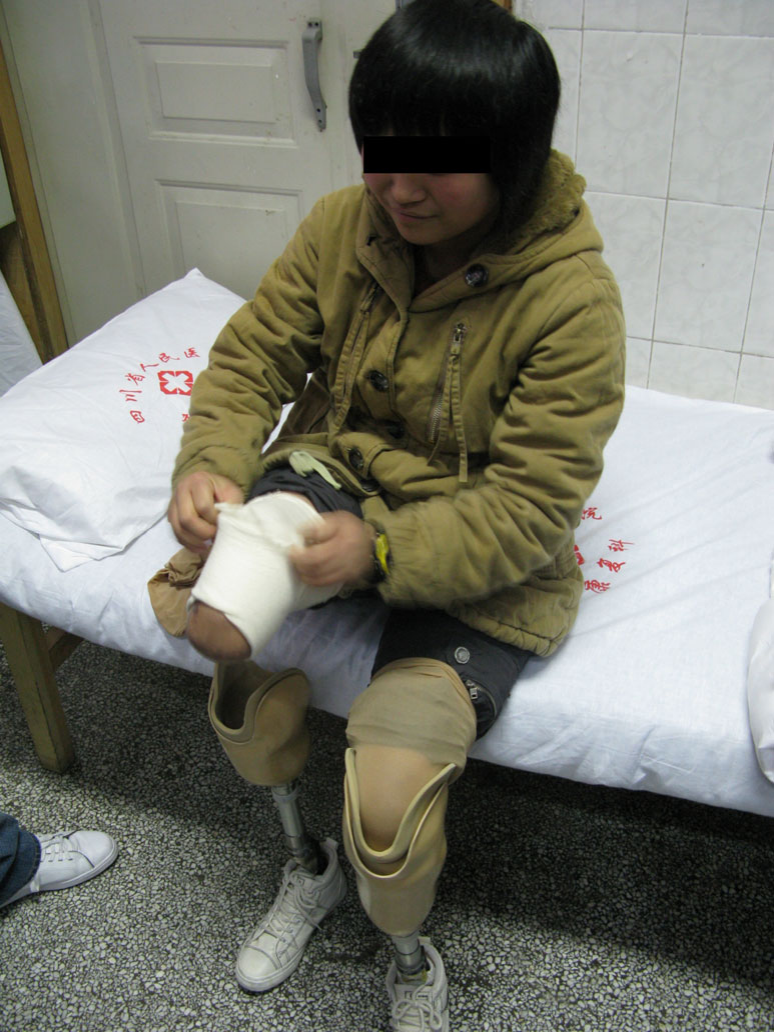
**The patient self donning and doffing her prostheses**.

The corresponding modification of the plaster cast was performed according to the information obtained through the transparent check socket. The definitive resin sockets were made of lamination. The alignment of the preparatory prostheses was transferred to the definitive prostheses. The fitting trial for the definitive prostheses was focused on gait characteristics. The goal was to set the prostheses to achieve a smooth, even and stable gait. The self-donning and doffing capability was another important issue (Figure 6).

In prosthetic fabrication, the communication with other rehabilitation team members was necessary to adjust the prosthetic setting to provide optimal fitting. The capability of functional improvement should be explored through the prostheses refinement process. The biomechanical factors, such as dynamic alignment, were adjusted to match her physical condition. By reviewing the progress of the patient, feedback and professional opinion from therapists, enhancement was suggested. Cosmetic foam covers were then added to restore the natural shape after the prostheses were finalized [22,23].

### 4. Prosthetic Training

At this stage, the patient has learned the basic principles behind the function of each of the components in the prostheses, their maintenance and care, and other points of prosthetic management. Despite this, use of the prostheses might cause pain in the residual limbs and the therapists must regularly check that she has put them on correctly and ensure proper fit [24].

The patient has practised how to don and doff the prostheses, how to determine the appropriate socks, and has acquired the techniques on how to adjust them. Skin care and inspection techniques were also reviewed. Transfer and weight shifting techniques were encouraged, including the use of steps and a balance board [25].

### 5. Functional Training

Gait training was integral to the rehabilitation process. It was essential that gait training initially addressed proper technique, following with endurance and velocity on flat surfaces [26,27]. Progressed to advanced skills training such as uneven terrains, elevations, stairs, curbs and ramps were also incorporated [28]. The patient has to be familiarize with various performances through repeated practices with and without using the prostheses. Dressing, toileting, bathing, and other daily activities were practiced regularly to maximize functional capabilities.

### 6. Community Reintegration

Reintegration into the community was best done as a gradual process. The therapists demonstrated and guided how the patient could accomplish skills in the community setting such as using escalators in shopping arcades, using public transportation, crossing traffic roads, and going on and off pavement [29].

Environmental modifications and assistive devices were introduced in order to achieve maximum independence at home, school and workplace. List of resources for information regarding amputations, support groups, and accessibility for people with disabilities were provided.

### 7. Recreational/Vocational Rehabilitation

The functional training should be specifically directed towards recreational and vocational goals. The rehabilitation team has provided education and information on recreation skills or resources, organizations with opportunities for adaptive recreational activities, long-term sport specific, prostheses or assistive devices available (e.g. specially designed prosthetic legs for running) [30].

Vocational rehabilitation and counseling should become part of the rehabilitation programme for those who are of working age. She can be referred to a vocational counselor for guidance regarding future vocational plans. It is crucial that vocation take place gradually, with time and workload increasing. Better cooperation between rehabilitation team members, professionals, implementing bodies, and the employers is necessary [31].

### 8. Long-Term Follow-Up

The patient should receive lifelong care and psychosocial adjustment to meet her current abilities, needs, goals and quality of life [32,33]. Regular follow-up should be provided to maintain the quality and functionality of the prosthetic limbs [34-36]. New technology should be considered but must be matched to her conditions and capability, and followed with an additional period of training to facilitate her in using the new components.

## Rehabilitation Outcomes

The patient's latest assessment and outcome evaluation were completed in the Prince of Wales Hospital, Hong Kong. There, the patient was invited to run on a treadmill, go through isokinetic and balance training. She has a much improved mood and was motivated to undergo exercise training and recreational activities. Her wounds had healed and the bilateral stumps were in good condition and shape. The phantom limb pain and sensitization was significantly reduced. She was no longer disturbed by phantom pain and scarring discomfort.

**Figure 7:**
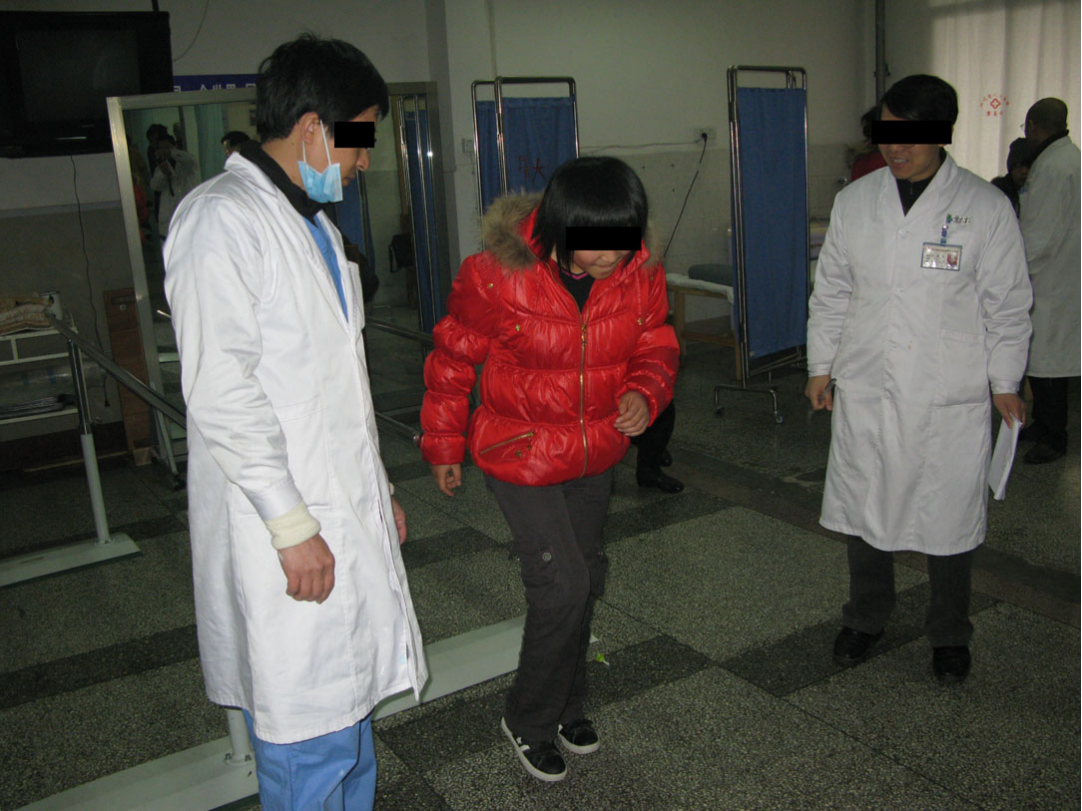
**Jumping activities training**.

**Figure 8:**
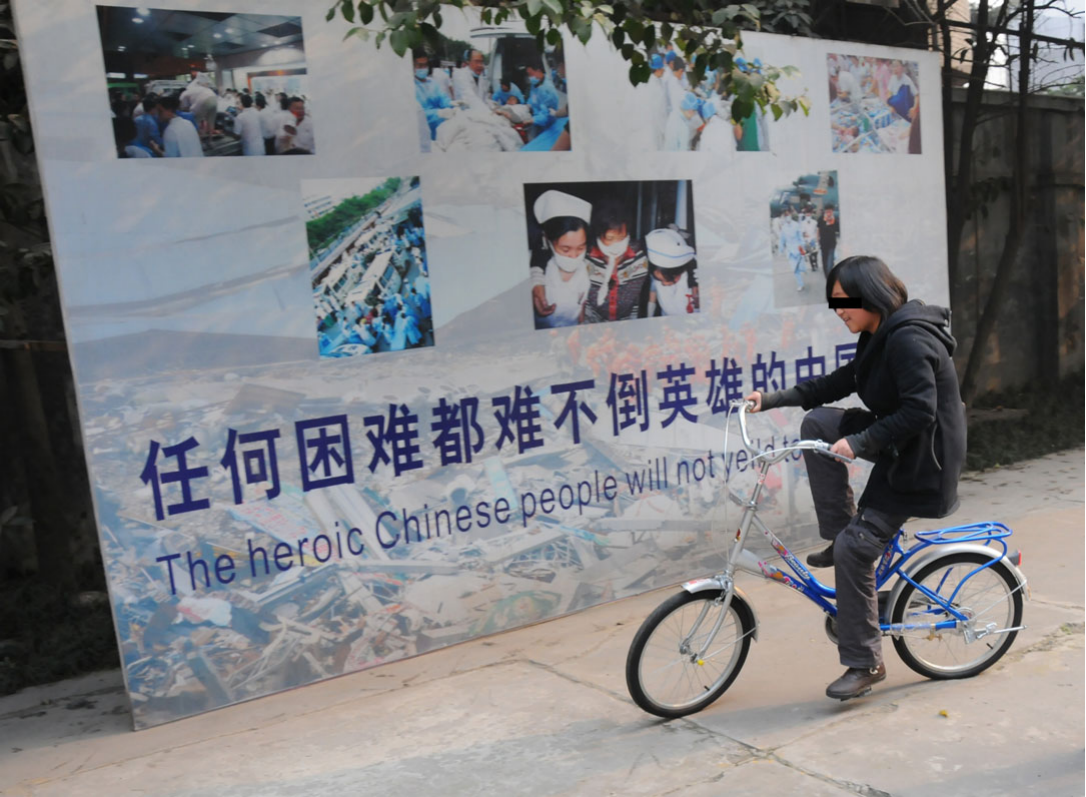
**Functional activities training**.

She has regained her joint mobility and muscle power. She was independent in transfers and could walk and even run independently on level and uneven ground. She could walk for hours and her facial expression showed no signs of fatigue. She managed stairs with ease, and has demonstrated high ability in balance and coordination. She has resumed her normal school life and participated in various outdoor activities (Figures 7, 8). She is satisfied with her condition and enjoys her new life. A long-term follow-up on body image and compliance of prosthetic use will be conducted periodically.

## Conclusion

This patient is a very active and optimistic girl. She has shown an extremely positive attitude with full participation throughout the rehabilitation process and her progress has been profound. Her positive attitude brightens up everyone around her. She has already returned to school with an active school life and effectively manages her daily activities. During the first year ceremony of the Sichuan earthquake, she was invited to come to Hong Kong by "Stand Tall", and was interviewed by various media outlets, including the international news agency CNN. There she presented her views on the injury and rehabilitation process.

Despite this catastrophic event that led to the injury of thoracolumbar vertebral collapse and bilateral limbs loss, early rehabilitation and specially designed bilateral prostheses successfully prepared her to stand again. The team approach of the medical and allied health staff working in a coordinated fashion is of considerable value in the rehabilitation process.

The joint efforts of the multidisciplinary team and the advancement of new technology have revolutionized the care process for amputees. The loss of a limb may not necessarily impair a person's opportunities; instead the motivated ones have more incentives in brightening their prospects and lives.

## Consent

Written informed consent was obtained from the patient for publication of this case report and accompanying images. A copy of the written consent is available for review by the Editor-in-Chief of this journal.

## Competing interests

The authors declare that they have no competing interests.

## Authors' Information

Caroline Ngar-Chi Wong holds the position of Physiotherapist in the Physiotherapy Department, Prince of Wales Hospital, P.R. China.

Joseph Man-Kit Yu holds the position of Prosthetist and Orthotist in the Prosthetic and Orthotic Department, Prince of Wales Hospital, P.R. China.

Sheung-Wai Law holds the position of Consultant in the Department of Orthopaedics and Traumatology, Prince of Wales Hospital, P.R. China.

Herman Mun-Cheung Lau holds the position of New Territories East Cluster Coordinator and Department Manager in the Physiotherapy Department, Prince of Wales Hospital, P.R. China.

Cavor Kai-Ming Chan holds the position of Chair Professor and Chief of Service in the Department of Orthopaedics and Traumatology, Prince of Wales Hospital, P.R. China.

## Authors' contributions

WNC and YMK involved in the rehabilitation program and prepared the manuscript. LSW and LMC coordinated the rehabilitation program. CKM initiated and coordinated the project and rehabilitation program. All authors contributed and approved the final manuscript.
